# Gut Microbiota in Schizophrenia: Taxonomic Shifts, Beta- Diversity Alterations, and Biomarker Potential: A Systematic Review

**DOI:** 10.3390/ijms27104606

**Published:** 2026-05-21

**Authors:** Andreea-Mihaela Militaru, Arina Cipriana Pietreanu, Simona Trifu, Gabriela Loredana Popa

**Affiliations:** 1 Department of Clinical Psychology, University of Bucharest, 050663 Bucharest, Romania; andreea-mihaela.militaru39@s.fpse.unibuc.ro; 2 Doctoral School, “Carol Davila” University of Medicine and Pharmacy, 020021 Bucharest, Romania; 3 Department of Neurosciences, “Carol Davila” University of Medicine and Pharmacy, 020021 Bucharest, Romania; 4 Department Infectious Diseases, Epidemiology, Microbiology, Parasitology, Virology, Diabetes, Endocrinology, “Carol Davila” University of Medicine and Pharmacy, 020021 Bucharest, Romania; gabriela.popa@umfcd.ro

**Keywords:** schizophrenia gut microbiome, gut-brain axis, meta-analysis, microbial dysbiosis

## Abstract

Emerging evidence implicates the gut–brain axis in the pathophysiology of schizophrenia, yet literature regarding specific microbiome alterations remains inconsistent. This study aims to synthesize evidence on gut microbiota diversity and taxonomic composition in individuals with schizophrenia compared to healthy controls. Unlike prior meta-analyses, this study integrates quantitative alpha diversity synthesis with cross-taxonomic qualitative analysis and contextualizes findings within functional frameworks of the gut–brain axis, highlighting the methodological heterogeneity that limits biological interpretation. A systematic review and meta-analysis were conducted following PRISMA 2020 guidelines. Electronic databases (Web of Science, PubMed, MDPI) were searched for observational studies published between 2017 and 2025. Forty-eight studies met inclusion criteria for qualitative synthesis, with 14 providing sufficient data for random-effects meta-analyses of alpha diversity. Meta-analyses revealed no statistically significant differences in alpha diversity indices (Shannon, Simpson, Chao1, ACE, Observed) between patients and controls, despite high heterogeneity. Conversely, beta diversity analyses generally demonstrated significant differences in microbial community composition. Taxonomic synthesis identified recurrent but heterogeneous dysbiotic patterns characterized by the depletion of short-chain fatty acid-producing taxa (e.g., *Faecalibacterium*, *Roseburia*, Lachnospiraceae) and enrichment of pro-inflammatory taxa (e.g., Proteobacteria, *Fusobacterium*). Schizophrenia is associated with evidence of compositional alterations and functional shifts rather than a global loss of microbial richness. These findings highlight candidate taxa that may warrant further investigation in biomarker-focused studies and microbiome-based therapeutics. However, these findings should be interpreted cautiously due to substantial heterogeneity and limited control for key confounders such as antipsychotic medication, diet, and life-style factors.

## 1. Introduction

Schizophrenia is a chronic, severe, and heterogeneous psychiatric disorder characterized by disturbances in thought processes, perception, emotion, and behavior. It is considered to be one of the most severe psychiatric disorders [1]. According to the Diagnostic and Statistical Manual of Mental Disorders, Fifth Edition (DSM-5) it typically involves positive symptoms such as delusions and hallucinations, negative symptoms including anhedonia, social withdrawal, and diminished emotional expression, as well as cognitive impairments affecting attention, memory, and executive functioning [2]. The disorder usually emerges in late adolescence or early adulthood and follows a lifelong course with fluctuating periods of exacerbation and low remission, high mortality rates and high global disease burden [3].

According to the Global Burden of Disease Study 2016 (GBD 2016), the global age-standardized point prevalence of schizophrenia is estimated at 0.28%, corresponding to approximately 20.9 million affected individuals [4]. This reflects an increase from an estimated 13.1 million cases in 1990, primarily driven by population growth rather than changes in individual risk.

More recent epidemiological assessments indicate that the global number of individuals living with schizophrenia has continued to rise, reaching approximately 24 million people worldwide, or about 1 in 300 adults, according to the World Health Organization [5].

In Europe, the estimated prevalence remains around 0.3%, though values vary across countries due to differences in diagnostic criteria, health-care system capacity, and methodological heterogeneity in epidemiological surveys (GBD 2019/2021 regional estimates).

Schizophrenia impairs multiple domains of a patient’s life, including significant deterioration in the quality of life in all areas: physical, social, psychological, environmental [6]. Individuals with schizophrenia often experience profound cognitive deficits, social withdrawal, and difficulties in maintaining employment or education, which collectively reduce their overall quality of life [7]. Additionally, the disorder is associated with increased comorbidity, including metabolic and cardiovascular conditions, and a higher risk of premature mortality compared to the general population [8]. These factors highlight schizophrenia not only as a clinical challenge but also as a substantial public health concern, underscoring the need for improved understanding of its underlying biological mechanisms.

The etiology of psychosis is believed to be multifactorial, arising from interactions between genetic and environmental factors [9,10,11] and the underlying biological mechanisms remain heterogeneous and poorly understood [12,13].

In particular, growing evidence has pointed to the role of the gut–brain axis and alterations in the intestinal microbiome as potential contributors to schizophrenia pathophysiology, immune dysregulation, metabolic disturbances, and comorbid somatic disease like gastrointestinal conditions such as celiac disease, colitis and irritable bowel syndrome [1,10,14,15,16,17].

The largest population of microorganisms on the human body resides in the gastrointestinal tract [18]. Known as the gut microbiota, this complex ecosystem is comprised of microorganisms including bacteria, fungi, and viruses from over 60 genera [19]. This ecosystem is linked with brain function, and psychosis itself is a condition that affects the way the brain processes information [20].

The gut–brain axis, a bidirectional communication network between the central nervous system and the gastrointestinal tract [10,21], mediates this interaction through multiple mechanisms: regulation of neurotransmitter synthesis (e.g., gamma-aminobutyric acid, serotonin, dopamine) [22].This axis encompasses major physiological systems: endocrine, nervous, immune, and metabolic, that collectively support and regulate behavioral responses [23]. Multiple processes have been explored as mechanisms supporting communication along this axis, including tryptophan metabolism [24], the hypothalamic–pituitary–adrenal axis [25], the generation of microbial metabolites such as short-chain fatty acids [26] and signaling through the vagus nerve [1], aspects also cited in Murray’s review (2023).

Alterations in these processes have been proposed to contribute to the pathophysiology of schizophrenia, potentially affecting symptom severity, cognitive function, and comorbid somatic conditions [14,27,28].

Several human studies have identified significant differences in gut microbiome composition between patients with schizophrenia and healthy controls [17,29]. Alpha diversity, reflecting the richness and evenness of microbial communities within an individual, is often reported as reduced in schizophrenia, although findings are sometimes inconsistent across cohorts [30,31,32,33]. Beta diversity, which quantifies compositional differences between individuals or groups, also appears altered in schizophrenia, suggesting a distinct microbial signature associated with the disorder [34].

Despite growing interest in the gut–brain axis and mounting studies investigating the gut microbiome in schizophrenia, the current literature remains plagued by considerable inconsistencies, methodological heterogeneity and important limitations, which justify the need for an updated, rigorous meta-analysis.

First, while some microbial taxa (e.g., Bifidobacterium, Lactobacillus, Megasphaera) appear more abundant in individuals with schizophrenia compared to controls [20], the evidence for consistent differences in alpha diversity (species richness, evenness) remains weak and statistically non-significant [31,35].

Furthermore, even when beta diversity differences (group-level compositional differences) are often reported, the environmental, demographic, and clinical confounders (e.g., diet, antipsychotic treatment, lifestyle, metabolic status) vary widely across studies, making it unclear whether observed microbiome variations reflect disease specific signatures or reflect external influences linked to patient status or there is lack of detailed dietary information [36].

Importantly, rather than attempting to identify statistically significant differences alone, this study aims to reconcile conflicting findings by distinguishing between global diversity metrics and compositional alterations across taxonomic levels, and by interpreting these findings in a functional and pathophysiological context. This approach al-lows a more nuanced understanding of microbiome alterations in schizophrenia beyond conventional diversity measures.

## 2. Materials and Methods

This systematic review and meta-analysis was conducted according to PRISMA 2020 guidelines. Eligibility criteria were defined according to the PICO framework to ensure consistency and relevance to the research question. This systematic review was not registered due to the exploratory nature and heterogeneity of available data.

The primary objective was to compile evidence of differences in fecal microbiota between patients with schizophrenia diagnosis and healthy individuals. Studies were included based on predefined eligibility criteria aligned with the objectives of this meta-analysis.

We included human observational studies, specifically case–control, cohort, and cross-sectional designs, that investigated gut microbiome characteristics in individuals diagnosed with schizophrenia. Eligible populations consisted of adults with a clinical diagnosis of schizophrenia according to standardized diagnostic criteria (e.g., DSM or ICD). Studies were required to include a healthy control group for comparison.

The intervention in this context refers to alterations in the gut microbiome associated with schizophrenia. Therefore, eligible studies must have investigated gut microbiota composition.

To be included, studies had to report at least one microbiome-related outcome, including: Alpha diversity indices (e.g., Shannon, Simpson, Chao1), Beta diversity metrics, or Taxonomic differences at any phylogenetic level (phylum to genus/species). Each alpha diversity index was analyzed and interpreted separately due to their distinct ecological properties (richness vs. evenness), and no pooled interpretation across indices was performed.

Only studies published in the last 5–7 years (2017–2025) and written in English were considered eligible.

We excluded: reviews, systematic reviews, and meta-analyses, animal studies or studies using rodent or other non-human models, studies not providing primary microbiome data, studies evaluating other psychiatric disorders (e.g., bipolar disorder, major depression, anxiety) unless data on schizophrenia were reported separately, conference abstracts, editorials, commentaries, and protocols lacking original data.

A comprehensive literature search was conducted across three major electronic databases: Web of Science, MDPI and PubMed. The search aimed to identify all relevant human observational studies evaluating gut microbiome alterations in individuals with schizophrenia. The final search was performed on December 2025. The search strategy was developed using a combination of controlled vocabulary and free-text terms related to schizophrenia and the gut microbiome. Boolean operators (“AND”, “OR”) and truncation were applied to broaden or narrow the search as appropriate. The search strings were customized for each database based on its indexing rules. For Web of Science we used the following search string: (“schizophrenia” OR “psychosis”) AND (“gut microbiome” OR “gut microbiota” OR “microbiome” OR “intestinal microbiota”).The search string for PubMed was: (“schizophrenia” [Mesh] OR “psychotic disorders” [Mesh] OR schizophrenia [tiab] OR psychosis [tiab]) AND (“gastrointestinal microbiome” [Mesh] OR “microbiota” [Mesh] OR “gut microbiome” [tiab] OR “gut microbiota” [tiab] OR microbiome [tiab] OR “intestinal microbiota” [tiab]) and for MDPI: schizophrenia OR psychosis AND “gut microbiome” OR “gut microbiota”.

All retrieved records were exported in compatible formats and imported into Rayyan for screening. Duplicate entries across the three databases were automatically detected and removed using Rayyan’s deduplication algorithm.

Two independent reviewers performed the assessment of all included studies to assess the internal validity and reliability of the evidence, using the Newcastle–Ottawa Scale (NOS) [37]. This tool assesses the quality of nonrandomized studies in systematic reviews and meta-analyses. The NOS evaluates studies based on three domains: Selection of study groups (maximum 4 stars), Comparability of groups (maximum 2 stars), Exposure/Outcome assessment (maximum 3 stars). Each study received a score reflecting its overall risk of bias: High quality (7–9 stars), Moderate quality (4–6 stars), Low quality (0–3 stars).

The assessment criteria for selection was: adequacy of case definition (confirmed schizophrenia diagnosis), representativeness of cases, selection of controls (healthy and comparable), definition and validation of control group. For comparability: adjustment for potential confounders affecting gut microbiome composition, like age, sex, BMI, medication status, diet. For exposure/outcome assessment: microbiome assessment methods (16S rRNA, shotgun metagenomics), blinding of laboratory personnel to case/control status, completeness of data reporting. Studies identified as high risk of bias were included in the qualitative synthesis.

All studies included in the meta-analysis met predefined quality criteria based on the Newcastle–Ottawa Scale (NOS). As no studies were identified as high risk of bias, sensitivity analyses excluding high-risk studies were not performed. Although formally assessed in the next section, studies at high risk of bias were considered when interpreting potential publication bias and heterogeneity in meta-analytic outcomes. Multiple complementary methods were used (Egger’s regression, Begg and Mazumdar tests, trim-and-fill, and p-uniform).

Data were managed and analyzed using Rayyan for screening and Excel for data extraction. Meta-analyses were conducted in Jamovi v1.6.

Data extracted from eligible studies were entered into a standardized Excel database and cross-checked for accuracy. Continuous outcomes, such as alpha diversity indices or taxonomic abundances, were recorded as mean ± standard deviation (SD) or median with interquartile range (IQR), where reported. When medians and IQRs were provided, data were converted to approximate means and SDs using established statistical methods. Categorical outcomes, when applicable, were coded consistently across studies. Missing or unclear data were addressed by contacting corresponding authors or estimating values from graphical representations using digital extraction tools for calibration. When numerical data were not reported, values were estimated from graphical representations using digital extraction tools. Although this approach is commonly used in me-ta-analyses, it may introduce measurement error and reduce precision, which was considered when interpreting results.

Meta-analyses were conducted using random-effects models to account for expected heterogeneity across studies in microbiome assessment methods, populations, and sequencing techniques. For continuous outcomes, the effect size was expressed as standardized mean difference (SMD) with 95% confidence intervals (CI). Random-effects meta-analysis with Knapp–Hartung adjustment was used. Forest plots including summary effects and prediction intervals were generated. Publication bias was visually assessed using contour-enhanced funnel plots. Pooled estimates were calculated separately for each outcome of interest, including alpha diversity.

Statistical heterogeneity was evaluated using: Cochran’s Q test (*p* < 0.10 considered significant) and I^2^ statistic, interpreted as: 0–25%: low heterogeneity, 26–50%: moderate heterogeneity, 51–75%: substantial heterogeneity, 75%: considerable heterogeneity. Due to inconsistent and incomplete reporting of key confounders (including anti-psychotic treatment, diet, BMI, and smoking status), subgroup analyses and me-ta-regression could not be reliably performed. Instead, these variables were qualitatively assessed during data extraction and considered in the interpretation of findings.

Potential publication bias was assessed for outcomes with ≥10 studies using: Funnel plots to visually inspect asymmetry and Egger’s regression test for statistical evaluation of small-study effects. If bias was detected, trim-and-fill analyses were performed to estimate its impact on pooled effect sizes. Multiple complementary methods were used: Egger’s regression, Begg and Mazumdar tests, trim-and-fill, and p-uniform.

## 3. Results

### 3.1. Study Selection and Characteristc

**Figure 1:**
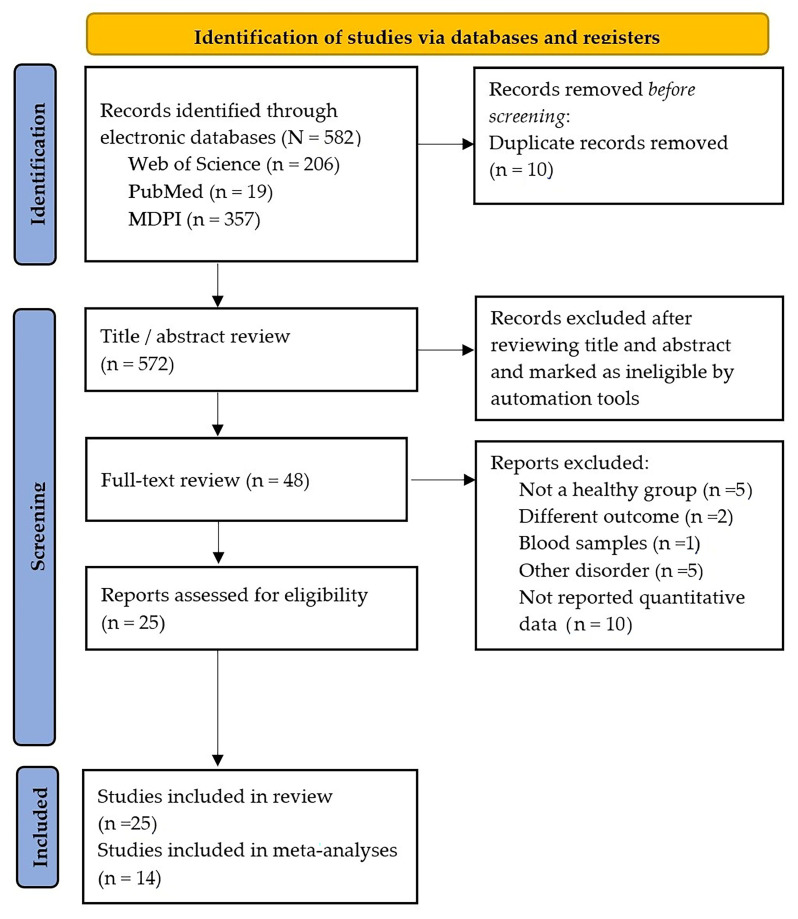
PRISMA flow diagram for studies of gut microbiome in schizophrenia and healthy control.

A total of 582 records were identified through database searching (Web of Science, PubMed, and MDPI). After removal of 10 duplicates, 572 records were screened based on title and abstract. Following full-text assessment, 48 studies met the eligibility criteria and were included in the qualitative synthesis, of which 14 studies provided sufficient data for quantitative meta-analysis (Figure 1).

The included studies in the meta-analyze were published between 2017 and 2025 and comprised a total of 861 schizophrenia patients and 719 healthy controls. Most studies employed a case–control, cross-sectional design and used 16S rRNA gene sequencing and only two used shotgun metagenomics to characterize the gut microbiome, in fecal samples. The alpha diversity indices included measures of community richness (Observed, Chao1, and ACE) and community diversity (Shannon, Simpson, Faith’s phylogenetic diversity (Faith_PD), and Evenness). These biomarkers were selected for their relevance to the pathology of SCZ and their consistency across studies.

Detailed characteristics are summarized in Supplementary Material.

Across individual studies, findings regarding alpha diversity were inconsistent across studies. Most of the studies reported no statistically significant differences between groups, while only three reported higher Faith’s phylogenetic in schizophrenia patients compared to healthy controls, whereas other three reported reduced microbial diversity in schizophrenia particularly for evenness-based indices (e.g., Shannon and Simpson). Most of the studies did not provide quantitative data for control confounders in treatment, diet, lifestyle). Given the high heterogeneity observed across most alpha diversity indices (I^2^ ranging from 83% to 96%), pooled estimates should be interpreted with caution, as they may reflect methodological and population variability rather than true biological absence of effect.

### 3.2. Qualitative Results

Qualitative analysis revealed distinct microbial abundance patterns in SCZ compared to healthy controls. (Supplementary Material).

Across the included studies, qualitative synthesis revealed relatively consistent but heterogeneous alterations in gut microbial composition in schizophrenia, spanning multiple taxonomic levels (phylum, family, genus, and species). Despite methodological heterogeneity, several recurrent patterns of enrichment and depletion emerged when comparing individuals with schizophrenia to healthy controls.

#### 3.2.1. Phylum-Level Alterations

At the phylum level, Bacteroidetes/Bacteroidota and Proteobacteria/Pseudomonadota were among the most frequently reported taxa showing increased abundance in schizophrenia, as observed across multiple studies [38,39,40,41]. Several studies also reported increases in Fusobacteria and Euryarchaeota in SCZ populations.

In contrast, Firmicutes/Bacillota were consistently reported as depleted in schizophrenia, with converging evidence from multiple independent cohorts [38,41,42]. Decreases in Verrucomicrobia and Tenericutes were also reported, though less consistently.

Schizophrenia may be characterized by a shift toward Proteobacteria/Fusobacteria dominance and reduced Firmicutes, suggesting a dysbiotic profile associated with inflammatory and metabolically unfavorable microbiota.

#### 3.2.2. Family-Level Alterations

At the family level, several short-chain fatty acid (SCFA) producing families were repeatedly reported as depleted in schizophrenia, including: Lachnospiraceae, Ruminococcaceae, Butyricicoccaceae, Coriobacteriaceae, Monoglobaceae. These families are primarily associated with butyrate production and gut barrier integrity, and their depletion was consistently observed across studies [39,40,43]. Conversely, potentially pro-inflammatory or opportunistic families were often enriched in SCZ, including: Enterobacteriaceae, Fusobacteriaceae, Campylobacteraceae, Desulfovibrionaceae, Erysipelotrichaceae, Turicibacteraceae. These taxa were more frequently reported in schizophrenia cohorts compared to controls [15,34,44].

Family-level alterations indicate a loss of beneficial SCFA-associated taxa and enrichment of inflammation-linked microbial families in schizophrenia.

#### 3.2.3. Genus-Level Alterations

The most robust qualitative findings emerged at the genus level, where several taxa showed relatively consistent directionality across studies.

The following genera were among the most repeatedly reported as reduced in SCZ: *Faecalibacterium*, Blautia, *Roseburia*, Coprococcus, Gemmiger, Agathobacter, Butyricicoccus, Lachnospira, Ruminococcus. These genera are well-known butyrate producers and markers of gut health. Their depletion was observed across numerous studies [39,43,45,46,47]. Conversely, schizophrenia was associated with increased abundance of several genera with pathobiontic or inflammatory potential, including: Proteus, Succinivibrio, Escherichia/Escherichia–Shigella, *Fusobacterium*, Desulfovibrio, Prevotella, Romboutsia, Streptococcus. These genera were repeatedly observed to be enriched in SCZ across independent cohorts [41,43,44,48].

At the genus level, schizophrenia is marked by a consistent depletion of SCFA-producing taxa alongside enrichment of potentially pathogenic and pro-inflammatory genera.

#### 3.2.4. Species-Level Findings

Although fewer studies reported species-level data, notable findings included increased abundance of: Bacteroides fragilis, Ruminococcus torques, *Fusobacterium* hominis, Desulfovibrio piger, Streptococcus gallolyticus and decreased abundance of: *Faecalibacterium* duncaniae, *Roseburia* hominis, Ligilactobacillus ruminis [10,43].

These species-level changes further support the loss of anti-inflammatory microbes and expansion of bacteria linked to epithelial disruption and immune activation.

### 3.3. Beta Diversity

Across the included studies, beta-diversity analyses consistently demonstrated significant differences in gut microbial community composition between individuals with schizophrenia (SCZ) and healthy controls (HCs). The majority of studies reported clear separation between SCZ and HC samples using multiple distance metrics, including Bray–Curtis dissimilarity, unweighted UniFrac, and weighted UniFrac, indicating robust alterations in overall microbial structure associated with schizophrenia.

#### 3.3.1. UniFrac-Based Beta Diversity

Most studies employing unweighted UniFrac distances, which assess phylogenetic differences based on the presence or absence of taxa, reported significant separation between SCZ and HC groups.

Specifically: several authors reported highly significant group differences (*p* ≤ 0.001) [32,34,39,42]. PCoA, PCA, and NMDS ordination plots consistently showed distinct clustering of SCZ and HC samples, indicating divergent microbial membership. Several studies [39,49] also observed greater within-group variability among SCZ patients, suggesting increased inter-individual heterogeneity in schizophrenia-associated microbiota. These findings suggest that schizophrenia is associated with altered phylogenetic composition of the gut microbiome, reflected in differences in microbial presence rather than solely abundance.

Studies using weighted UniFrac distances, which additionally account for relative abundance, similarly demonstrated significant β-diversity differences between SCZ and HCs. For example: Chen et al. (2023) [50], Chen et al. (2024) [40], Nita et al. (2025) [46], and Tao et al. (2025) [44] reported significant separation (*p* values ranging from 0.049 to <0.001), with schizophrenia diagnosis explaining a non-trivial proportion of variance in microbiome composition (R^2^ values ranging from ~1.5% to 15%). Importantly, Nita et al. [46] showed that schizophrenia status remained a significant contributor to β-diversity even after adjusting for confounders such as smoking, diet, lifestyle, metabolic variables, and comorbidities, although the explained variance was attenuated (adjusted R^2^ ≈ 4.4%). Overall, weighted UniFrac results indicate that schizophrenia-related microbiome alterations involve changes in both phylogenetic structure and relative abundance of shared taxa.

#### 3.3.2. Bray–Curtis

Bray–Curtis–based analyses consistently supported UniFrac findings, revealing significant compositional differences between SCZ and HC microbiomes.

Notable results include significant PERMANOVA results reported by several researchers [32,40,41,44,45,46,49,50]. R^2^ values from Bray–Curtis analyses typically ranged between ~1.5% and 5%, indicating modest but statistically robust effects. These studies observed distinct clustering of SCZ samples in ordination space, often accompanied by greater dispersion relative to healthy controls. These results indicate that schizophrenia is associated with quantitative shifts in microbial community composition, not merely taxonomic turnover.

While most studies reported significant β-diversity differences, a small number highlighted the role of confounding variables: Rust found that after adjusting for sex, smoking, and alcohol consumption, overall β-diversity differences between SCZ and HC were no longer significant, although smoking independently contributed to microbiome variance [51]. Several studies did not report β-diversity analyses [38,47,52,53,54]. These findings suggest that lifestyle factors may partially modulate microbiome differences, but do not fully account for the consistent β-diversity separation observed across the majority of studies.

### 3.4. Quantitative Synthesis (Meta-Analysis)

Random-effects meta-analyses were conducted for each alpha diversity index due to expected methodological and clinical heterogeneity across studies. Sensitivity analyses (e.g., leave-one-out analysis) and subgroup analyses were not per-formed due to the limited number of studies available for each outcome, which would have resulted in unstable and potentially misleading estimates.

It was possible to ascertain means and standard deviations from 14 studies. These were included in the meta-analysis (861 cases and 719 controls). Indices used to assess alpha diversity included: Indices of richness (Chao 1, Ace, Observed Species), indices of evenness (Shannon, Simpson) and indices of biodiversity (Faith’s Phylogenetic Diversity) see Table 1. The most commonly used were Shannon and Simpson. Due to the fact that only one study reported Faith’s Phylogenetic Diversity, we couldn’t insert it into our meta-analysis. A summary of the random-effects meta-analyses for all alpha diversity indices is presented in Table 1. It summarizes the pooled effect sizes, heterogeneity estimates, and sample sizes for all alpha diversity indices included in the meta-analyses.

#### 3.4.1. Shannon Alpha Diversity

A random-effects meta-analysis including 13 studies examined differences in Shannon alpha diversity between individuals with schizophrenia and healthy controls. The pooled effect size indicated no statistically significant difference in Shannon diversity between schizophrenia patients and healthy controls (SMD = −0.12, SE = 0.13, z = −0.96, *p* = 0.36, 95% CI [−0.40, 0.16]). This result suggests that, when averaged across studies, Shannon alpha diversity did not differ significantly between groups. Although the pooled estimate suggested a trend toward lower Shannon diversity in schizophrenia, this difference did not reach statistical significance (Table 2 and Supplementary Material).

Substantial heterogeneity was observed across studies (τ^2^ = 0.18, τ = 0.42, I^2^ = 83.21%, Q(12) = 76.91, *p* < 0.001). The high I^2^ value indicates that more than 80% of the observed variance in effect sizes was attributable to between-study heterogeneity rather than sampling error, reflecting differences in study populations, microbiome sequencing methodologies, and analytical pipelines. Publication bias was assessed using multiple complementary approaches.

**Figure 2:**
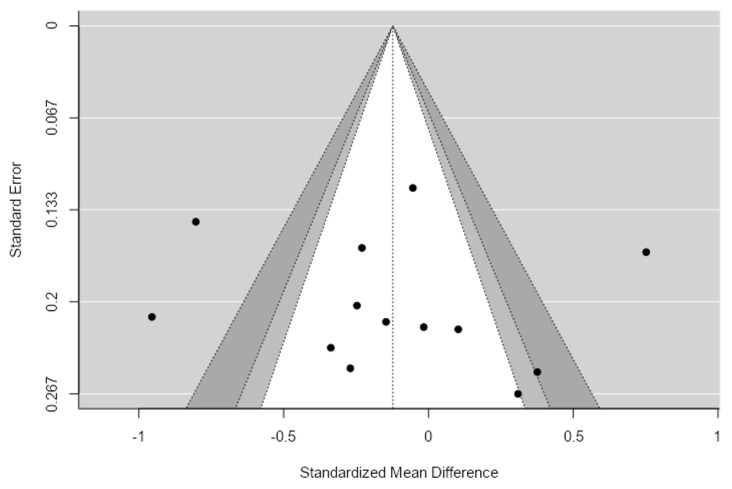
Shannon Funnel Plot.

The Egger’s regression test (*p* = 0.61) and Begg and Mazumdar rank correlation test (*p* = 0.20) did not indicate significant funnel plot asymmetry. Additionally, the trim-and-fill procedure suggested that no studies were missing due to publication bias. Although the fail-safe *N* was modest (*N* = 17), indicating that a relatively small number of unpublished null studies could change the overall significance, the absence of funnel plot asymmetry suggests that publication bias is unlikely to have materially affected the pooled estimate (Figure 2).

#### 3.4.2. Simpson Index

For the Simpson index, the random-effects meta-analysis included eleven studies. The results showed no significant difference in alpha diversity between patients with schizophrenia and healthy controls, SMD = −0.03, 95% CI [−0.39, 0.34], *p* = 0.87. The confidence interval included zero, indicating the absence of a consistent association between schizophrenia and Simpson diversity. The analysis revealed high heterogeneity across studies, I^2^ = 87.24%, Q(10) = 75.48, *p* < 0.001, suggesting substantial variability in effect estimates between studies. Assessment of publication bias did not indicate clear evidence of funnel plot asymmetry. Both the Begg and Mazumdar test (*p* = 0.087) and Egger’s regression test (*p* = 0.179) were non-significant. The trim-and-fill analysis estimated the addition of two hypothetical studies, which did not substantially alter the overall effect estimate (Table 3 and Supplementary Material).

**Figure 3:**
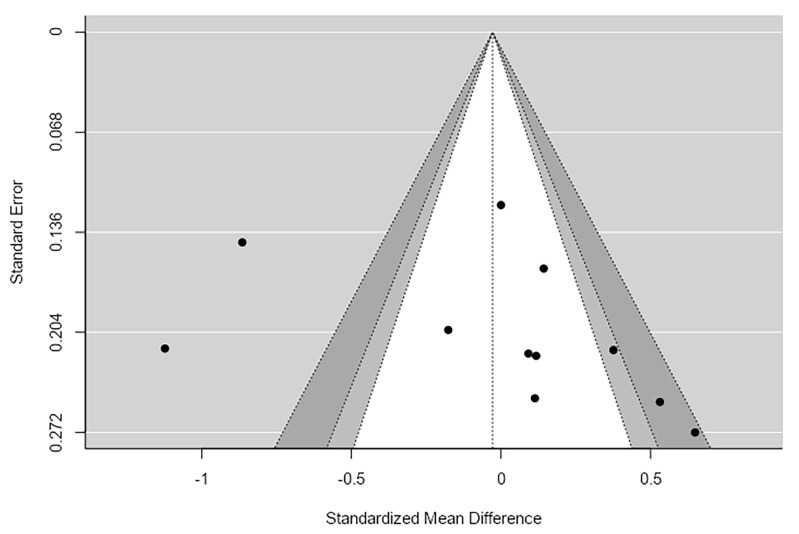
Simpson Funnel Plot.

The p-uniform analysis was conducted to examine potential publication bias and to estimate the overall effect size. The p-uniform publication bias test indicated no evidence of bias, test statistic = −0.256, *p* = 0.601. The estimated overall effect size was very close to zero, g = 0.017, 95% CI [−1.802, 0.768], with only 2 studies reaching statistical significance. This suggests that the effect of interest is negligible and inconsistent across studies. Equivalence testing using Two One-Sided Tests (TOST) further supported the absence of a meaningful effect. Both bounds were significant (lower Z = 2.896, *p* = 0.002; upper Z = −3.240, *p* = 0.001), and the equivalence confidence interval ranged from −0.296 to 0.240, indicating that the observed effect is statistically equivalent to zero. Overall, the p-uniform analysis suggests that there is no substantial effect and no evidence of publication bias for the outcome analyzed (Figure 3).

#### 3.4.3. Observed Alpha Diversity

A random-effects meta-analysis was conducted across eight studies (k = 8) to examine differences in Observed alpha diversity between patients with schizophrenia and healthy controls. The pooled effect size estimate was g = −0.168, SE = 0.096, which was not statistically significant (Z = −1.75, *p* = 0.123, 95% CI [−0.395, 0.059]) (Table 4 and Supplementary Material).

Heterogeneity statistics indicated moderate variability among studies I^2^ = 42.58%, Q(7) = 11.905, *p* = 0.104. These values suggest that some differences in effect sizes exist across studies, but the heterogeneity is not statistically significant.

**Figure 4:**
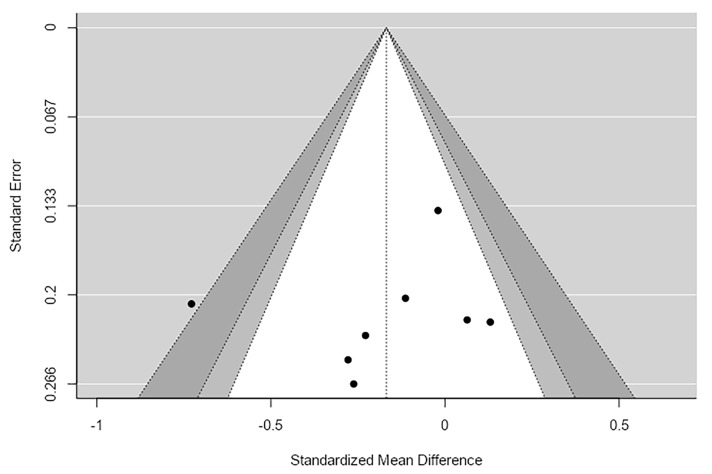
Observed Funnel Plot.

Several methods were used to assess potential publication bias. Assessment of publication bias did not indicate clear evidence of funnel plot asymmetry. Both the Begg and Mazumdar test (*p* = 0.399) and Egger’s regression test (*p* = 0.586) were non-significant. The trim-and-fill analysis did not impute any studies, and the p-uniform analysis confirmed a negligible effect size (g = 0.000, *p* = 0.990). Equivalence testing using Two One-Sided Tests further supported that the observed effect was statistically equivalent to zero (Figure 4).

#### 3.4.4. ACE Alpha Diversity

For the ACE index, the random-effects meta-analysis included seven studies. The results showed no significant difference in alpha diversity between patients with schizophrenia and healthy controls, SMD = −0.422, 95% CI [−1.450, 0.605], *p* = 0.353. The confidence interval included zero, indicating the absence of a consistent association between schizophrenia and ACE diversity (Table 5 and Supplementary Material).

**Figure 5:**
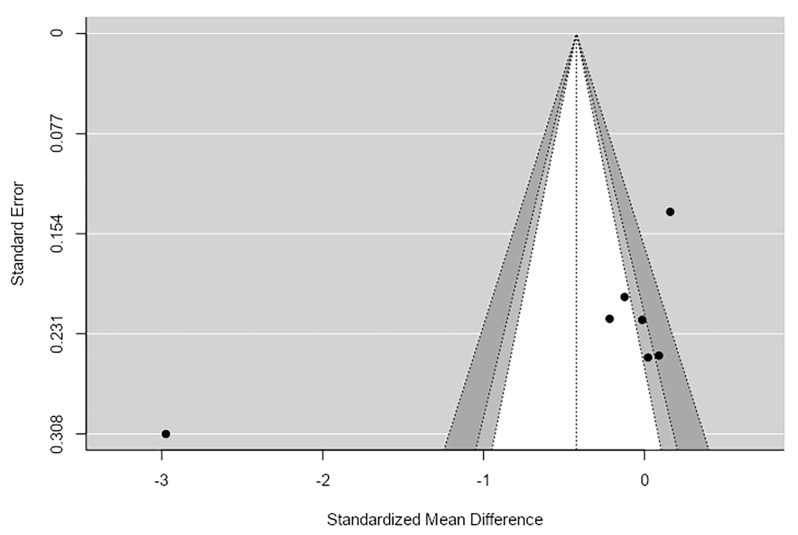
ACE Funnel Plot.

The analysis revealed very high heterogeneity across studies, I^2^ = 96.18%, Q(6) = 91.245, *p* < 0.001, suggesting substantial variability in effect estimates between studies. Assessment of publication bias did not indicate clear evidence of funnel plot asymmetry. Both the Begg and Mazumdar test (*p* = 0.562) and Egger’s regression test (*p* = 0.078) were non-significant. The trim-and-fill analysis did not impute any studies, and the p-uniform analysis confirmed a negligible effect size (g = 0.000, *p* = 0.990). The Test of Excess Significance showed one observed significant study versus seven expected, with an observed/expected ratio of 0.202. Equivalence testing using Two One-Sided Tests further suggested that the observed effect was statistically equivalent to zero, with an equivalence interval of [−1.113, 0.268] (Figure 5).

#### 3.4.5. CHAO1 Alpha Diversity

For the Chao index, the random-effects meta-analysis included seven studies. The results showed no significant difference in alpha diversity between patients with schizophrenia and healthy controls, SMD = −0.073, 95% CI [−0.221, 0.075], *p* = 0.274. The confidence interval included zero, indicating the absence of a consistent association between schizophrenia and Chao diversity (Table 6 and Supplementary Material).

**Figure 6:**
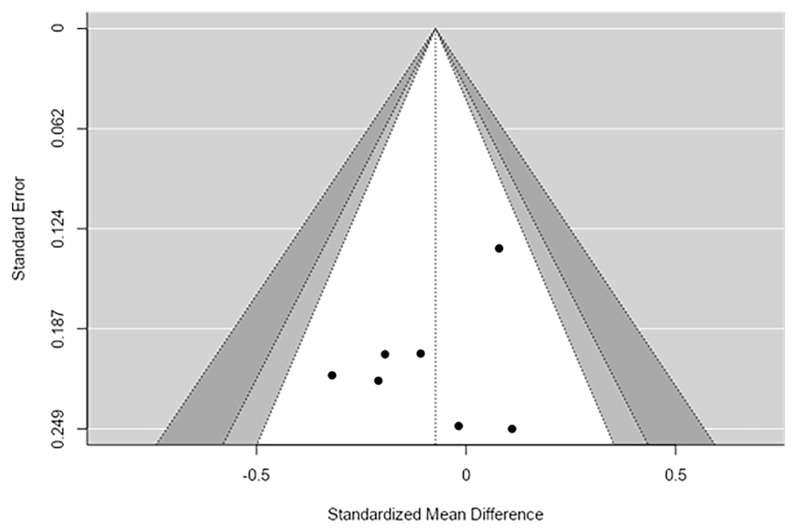
Chao1 Funnel Plot.

The analysis revealed no heterogeneity across studies, I^2^ = 0%, Q(6) = 3.901, *p* = 0.690, suggesting that effect estimates were highly consistent between studies. Assessment of publication bias did not indicate clear evidence of funnel plot asymmetry. Both the Begg and Mazumdar test (*p* = 1.000) and Egger’s regression test (*p* = 0.324) were non-significant. The trim-and-fill analysis did not impute any studies, and the p-uniform analysis confirmed a negligible effect size (g = 0.000, *p* = 0.990). The Test of Excess Significance showed zero observed significant studies versus seven expected, with an observed/expected ratio of 0.000. Equivalence testing using Two One-Sided Tests further supported that the observed effect was statistically equivalent to zero, with an equivalence interval of [−0.173, 0.027] (Figure 6).

## 4. Discussion

This systematic review and meta-analysis synthesized quantitative and qualitative evidence on gut microbiome alterations in schizophrenia. Meta-analyses of each individual alpha-diversity indices (Shannon, Simpson, Chao1, ACE, and Observed species) showed no consistent differences between individuals with schizophrenia and healthy controls, despite substantial between-study heterogeneity. In contrast, qualitative synthesis of taxonomic findings revealed relatively consistent but heterogeneous patterns of microbial alterations in schizophrenia, characterized by depletion of short-chain fatty acid–producing taxa and enrichment of potentially pro-inflammatory and pathogenic bacteria. Also, beta-diversity analyses consistently demonstrated significant differences in gut microbial community composition between individuals with schizophrenia (SCZ) and healthy controls (HCs), providing moderate evidence that schizophrenia is associated with a distinct and heterogeneous gut microbial community structure compared to healthy populations.

In the present meta-analysis, no consistent differences were observed across individual alpha diversity indices between patients with schizophrenia and healthy controls. These results suggest that, despite the known pathophysiological alterations in schizophrenia, overall, within-sample microbial diversity remains largely unchanged. This finding aligns with previous meta-analytic work [20,38], which similarly reported nonsignificant differences in alpha diversity metrics between schizophrenia patients and controls. This supports the absence of a global loss of microbial richness or evenness, but rather with compositional and functional shifts within the gut microbiome.

Several factors may explain these observations. First, alpha diversity indices summarize overall richness and evenness, which may remain relatively stable even when specific taxa are altered. Second, methodological variability across studies, such as differences in extraction, sequencing regions, and analytical pipelines, may mask subtle group differences.

In contrast to alpha diversity, taxonomic composition showed recurrent but heterogeneous alterations across multiple levels. At the phylum level, schizophrenia was associated with increased Proteobacteria, Fusobacteria, and Euryarchaeota and decreased Firmicutes, reflecting a dysbiotic and pro-inflammatory gut profile. At the family and genus levels, beneficial SCFA-producing taxa (e.g., Lachnospiraceae, Ruminococcaceae, *Faecalibacterium*, *Roseburia*) were depleted, while potentially pathogenic or pro-inflammatory genera (e.g., Enterobacteriaceae, *Fusobacterium*, *Desulfovibrio*) were enriched. These findings suggest that schizophrenia is associated with specific compositional shifts, even when overall alpha diversity remains unchanged. The consistent depletion of *Faecalibacterium*, *Roseburia*, *Blautia*, and *Coprococcus* aligns with evidence linking schizophrenia to impaired butyrate production, low-grade inflammation, and altered immune signaling. Our results are in agreement with prior literature showing depletion of *Faecalibacterium*, *Roseburia* [50], depletion on Firmicutes [30], increased Bacteroidetes [55]. In contrast of out results, Tamburini found increased abundance of Proteobacteria [56].

The meta-analysis also revealed high heterogeneity for several indices, notably Shannon (I^2^ = 83.2%), Simpson (I^2^ = 87.2%), and ACE (I^2^ = 96.2%). This substantial variability likely reflects differences in study populations, sequencing methods, analytical approaches, geographic location, dietary patterns, and antipsychotic treatment status. However, this level of heterogeneity substantially weakens the interpretability of pooled estimates and limits confidence in the absence of observed effects. In contrast, Chao1 and Observed indices showed lower heterogeneity (I^2^ = 0–42%), indicating more consistent estimates for richness measures.

Beta diversity analyses consistently demonstrated significant differences in microbial composition between schizophrenia patients and healthy controls. Both unweighted and weighted UniFrac, as well as Bray–Curtis metrics, indicated statistically significant but modest separation of samples, with schizophrenia explaining a modest but statistically significant proportion of variance (R^2^ ≈ 1.5–15%). This suggests that schizophrenia is associated with altered phylogenetic structure and relative abundance of key taxa, even in the absence of global alpha diversity changes. Recent evidence suggests that schizophrenia is not associated with changes in alpha diversity, but significant differences in beta diversity are consistently observed between patients and controls [36,49,57,58]. Importantly, these findings should be interpreted as associations rather than evidence of disease-specific microbiome signatures, given the lack of control for major confounding variables.

In other words, the overall microbial ecosystem size and diversity remain relatively stable. Interestingly, this pattern differs from other psychiatric disorders such as major depressive disorder, where reduced alpha diversity has been more consistently reported, suggesting that schizophrenia may be characterized more by compositional shifts rather than global diversity loss [59,60]. This distinction may indicate disorder-specific microbiome signatures, where schizophrenia appears to be characterized more by compositional shifts rather than a uniform loss of microbial diversity.

Importantly, alpha diversity indices capture distinct ecological dimensions of the microbiome. Shannon and Simpson indices reflect both richness and evenness, whereas Chao1 and ACE primarily estimate species richness. Observed species directly quantify detected taxa without accounting for unseen diversity. Therefore, the absence of consistent differences across these indices suggests that neither richness nor evenness is systematically altered in schizophrenia, although variability across metrics indicates potential subtle or context-dependent effects.

However, the observed compositional shifts specifically, the depletion of beneficial SCFA-producing genera such as *Faecalibacterium*, *Roseburia*, *Blautia*, and *Coprococcus* suggest functional consequences. These taxa are key producers of butyrate, a short-chain fatty acid critical for maintaining intestinal barrier integrity, anti-inflammatory signaling, and modulation of the gut-brain axis. Their depletion may lead to lower butyrate levels, promoting low-grade systemic inflammation, altered immune responses, and potentially contributing to schizophrenia-related pathophysiology, including neurotransmitter dysregulation and neuroinflammation.

In addition to the depletion of SCFA-producing taxa, the enrichment of potentially pro-inflammatory genera such as *Proteus* and *Fusobacterium* may contribute to immune dysregulation in schizophrenia. *Proteus* are capable of eliciting strong immune responses in humans through their lipopolysaccharides (LPS), which activate complement path-ways and induce pro-inflammatory cytokine production, and have been shown to adhere to and invade epithelial cells in intestinal disease contexts [61,62]. Similarly, Gram-negative taxa such as *Fusobacterium* are well recognized for their LPS-mediated pro-inflammatory activity and their ability to promote immune activation and dysbiosis [63]. Increased circulating LPS has been shown to disrupt intestinal tight junctions and increase gut permeability, thereby facilitating systemic inflammation [64]. Moreover, microbiome alterations characterized by enrichment of pro-inflammatory taxa have been linked to elevated peripheral cytokines and to structural and functional brain changes in patients with schizophrenia, suggesting that microbiota-driven inflammation may represent a key mechanism underlying gut–brain axis dysfunction [35,65].

Metrics such as unweighted and weighted UniFrac and Bray–Curtis show that schizophrenia is associated with reorganization of the microbial community, meaning that certain taxa are enriched (e.g., *Proteus*, *Fusobacterium*, *Desulfovibrio*) while others are depleted. This altered phylogenetic structure and relative abundance can affect microbial metabolic outputs, immune signaling, and gut-brain interactions. Even though the total number of taxa is similar, the functional potential of the microbiome is shifted, reflecting a dysbiotic profile that may influence inflammation, neurotransmission, and metabolic pathways relevant to schizophrenia.

In other words, schizophrenia does not reduce the overall number or diversity of gut microbes, but it changes who is there and in what proportions, leading to functional consequences for gut-brain communication and immune regulation.

This meta-analysis offers several methodological strengths. Integration of meta-analytic quantitative synthesis with qualitative taxonomic and beta diversity analyses allowed a comprehensive evaluation of microbiome alterations. Study selection was conducted using the Rayyan platform, ensuring systematic and unbiased inclusion/exclusion decisions. All meta-analyses used random-effects models to account for between-study heterogeneity, enhancing generalizability. Multiple complementary methods (Egger’s regression, Begg and Mazumdar tests, trim-and-fill, and p-uniform) suggested that publication bias was unlikely to materially affect pooled estimates. The analysis spanned phylum, family, genus, and species levels, providing a robust and biologically informative characterization of schizophrenia-associated dysbiosis. Additional strengths include the inclusion of recent studies (2017–2025), covering diverse populations, and the focus on 16S rRNA sequencing with uniform fecal sampling, ensuring comparability across cohorts.

### 4.1. Limitations of the Evidence Included

Despite these strengths, several limitations in the included studies should be noted.

A major limitation of this study is the inability to control for key confounders such as antipsychotic medication, diet, BMI, and smoking; no subgroup analyses or meta-regression could be performed to explore sources of heterogeneity due to insufficient reporting of confounding variables across studies.

Consequently, it remains difficult to disentangle disease-related microbiome alterations from medication-induced effects. This limitation further restricts the ability to identify microbiome signatures specific to first-episode psychosis or medication-naïve patients, who may exhibit distinct microbial profiles prior to pharmacological exposure. Although most studies indicated that patients were receiving psychiatric medication, de-tails regarding specific agents, dosage, and treatment duration were often lacking. These factors are known to substantially influence gut microbiome composition and may ac-count for a significant proportion of the observed variability. As a result, the findings should be interpreted as associative rather than disease-specific, and causal inferences cannot be made. 

The majority of studies were observational and cross-sectional, preventing causal inference. Differences in alpha diversity indices, LDA thresholds, and analytical pipelines contributed to heterogeneity. The very high heterogeneity observed across studies substantially limits the interpretability of pooled estimates. The absence of significant differences in alpha diversity may therefore reflect statistical noise and methodological variability rather than a true lack of biological effect. Many studies reported results without mean ± SD values, requiring data extraction from plots, calibrating them, which may introduce estimation errors and reduce precision. Some studies had small cohorts that potentially can reduce statistical power. The absence of sensitivity analyses (e.g., leave-one-out) limits the ability to assess the robustness of pooled estimates, and therefore findings should be interpreted with caution. 

Referring to the review process, not all eligible studies could be included in the meta-analysis due to insufficient quantitative data, particularly for alpha diversity. Variability in reporting standards and lack of uniform confounder adjustment (e.g., BMI, diet, lifestyle, treatment) limited the ability to perform moderator or subgroup analyses. Only 14 out of 48 eligible studies provided sufficient data for quantitative alpha diversity meta-analysis.

The potential use of microbiome alterations as biomarkers remains speculative, as this study did not include predictive modeling, validation cohorts, or diagnostic accuracy assessments.

### 4.2. Implications for Future Research

Future investigations should aim to conduct longitudinal studies to assess temporal dynamics of microbiome alterations in schizophrenia. Standardize reporting with mean ± SD for alpha diversity indices to facilitate meta-analytic synthesis. Integrate multi-omics approaches (metagenomics, metabolomics, metatranscriptomics) to elucidate functional implications. Control for confounders such as diet, BMI, medication, smoking, and lifestyle factors. Evaluate interventions including pre- and probiotics in randomized controlled trials to test causality and therapeutic potential.

Schizophrenia-associated microbiome alterations may represent candidate targets for future biomarker research, pending validation in appropriately designed studies. Targeted interventions to restore SCFA-producing taxa could support gut barrier integrity and reduce inflammation. Nutritional and microbiome-based strategies may complement conventional pharmacological treatment, potentially improving treatment response and symptom management. Understanding beta diversity differences can inform precision medicine approaches, identifying patients with more pronounced microbial dysbiosis for tailored interventions.

Taken together, these findings highlight the importance of cautious interpretation of microbiome meta-analyses in psychiatry, where methodological variability and con-founding factors remain substantial challenges.

In summary, our meta-analysis demonstrates that while individual alpha diversity indices largely unchanged in schizophrenia, there are suggestive but heterogeneous taxonomic alterations accompanied by significant beta diversity alterations. These findings highlight the importance of considering both taxonomic composition and functional capacity of the gut microbiome in schizophrenia, while future studies should aim to reduce methodological variability and explore causality.

## 5. Conclusions

This meta-analysis indicates that alpha diversity of the gut microbiome does not differ significantly between individuals with schizophrenia and healthy controls, as reflected across Shannon, Simpson, ACE, Observed, and Chao1 indices. Nevertheless, recurrent but not fully consistent taxonomic alterations were observed, including depletion of SCFA-producing and beneficial genera and enrichment of potentially pro-inflammatory taxa. In addition to SCFA depletion, enrichment of taxa such as Proteus and *Fusobacterium* may contribute to increased intestinal permeability, endotoxin production (e.g., lipopolysaccharides), and systemic immune activation. These processes have been implicated in neuroinflammation and may influence neurotransmitter systems relevant to schizophrenia. Beta diversity analyses further confirm consistent but modest and heterogeneous differences in microbial community composition between schizophrenia patients and controls. These findings highlight a pattern of dysbiosis characterized by compositional rather than global diversity changes, with potential relevance for future biomarker research and hypothesis generation and microbiome-targeted interventions. Future research should focus on longitudinal designs, standardized reporting, multi-omics integration, and con-trolled trials to clarify causal relationships and therapeutic opportunities in schizophrenia.

## Figures and Tables

**Table 1:** Summary of random-effects meta-analyses of gut microbiota alpha diversity indices in schizophrenia and healthy controls.

Biomarkers	No. Studies	N_SCZ	N_HC	*p*-Value	I^2^	SMD (95% CI)
Shannon	13	861	719	0.357	83.21%	−0.12 [−0.40, 0.16]
Simpson	11	699	623	0.867	87.24%	−0.03 [−0.39, 0.34]
ACE	7	350	356	0.353	96.18%	−0.42 [−1.45, 0.60]
Observed	8	397	386	0.123	42.58%	−0.17 [−0.40, 0.06]
Chao1	7	360	361	0.274	0%	−0.07 [−0.22, 0.08]

**Table 2:** Shannon index.

Study	Alpha Index	1	2	3	4	5	6	7	Std. Mean Difference IV, Random, 95% CI
Shi et al., 2023 [10]	Shannon	39	3.08	0.73	44	3.01	0.62	7.48%	0.10 [−0.33, 0.53]
Chen et al., 2024 [40]	Shannon	30	3.48	0.72	36	3.63	0.35	7.07%	−0.27 [−0.76, 0.22]
Chen et al., 2021 [42]	Shannon	63	4.27	0.76	40	4.44	0.54	7.73%	−0.25 [−0.64, 0.15]
Yan et al., 2022 [39]	Shannon	50	4.33	0.52	50	4.85	0.56	7.61%	−0.95 [−1.37, −0.54]
Nita et al., 2025 [46]	Shannon	35	3.54	0.31	35	3.42	0.45	6.80%	0.31 [−0.21, 0.83]
Chen et al., 2025 [34]	Shannon	35	3.19	0.6	30	2.96	0.61	7.03%	0.38 [−0.12, 0.87]
Wu et al., 2025 [45]	Shannon	87	3.27	0.13	70	3.3	0.13	8.30%	−0.23 [−0.55, 0.09]
Wu et al., 2024 [41]	Shannon	182	3.29	0.21	120	3.3	0.14	8.83%	−0.05 [−0.28, 0.18]
Zhu et al., 2017 [23]	Shannon	40	3.14	0.64	44	3.15	0.58	7.50%	−0.02 [−0.44, 0.41]
Zhu et al.,2025 [43]	Shannon	36	4.3	0.38	55	4.39	0.72	7.56%	−0.15 [−0.57, 0.27]
Shao et al., 2023 [47]	Shannon	90	7.1	1.05	71	6.43	0.62	8.26%	0.75 [0.43, 1.07]
Yuan et al., 2019 [29]	Shannon	107	5.14	0.92	107	5.76	0.58	8.55%	−0.80 [−1.08, −0.52]
Vasileva et al., 2024 [54]	Shannon	72	0.047	0.41	25	0.18	0.33	7.29%	−0.34 [−0.79, 0.12]
	**Total (95% CI)**	**866**			**727**			**100%**	**−0.12 [−0.40, 0.16]**

Legend: 1—N_SCZ, 2—mean_Shannon_SCZ, 3—SD_Shannon_SCZ, 4—N_HC, 5—mean_Shannon_HC, 6—SD_Shannon_HC, 7—Weight.

**Table 3:** Simpson’s Evenness Index.

Study	Alpha Index	1	2	3	4	5	6	7	Std. Mean Difference IV, Random, 95% CI
Shi et al., 2023 [10]	Simpson	39	3.08	0.73	44	3	0.62	8.94%	0.12 [−0.31, 0.55]
Chen et al., 2024 [40]	Simpson	30	0.09	0.08	36	0.06	0.02	8.51%	0.53 [0.04, 1.02]
Chen et al., 2021 [42]	Simpson	63	0.89	0.06	40	0.9	0.05	9.17%	−0.18 [−0.57, 0.22]
Yan et al., 2022 [39]	Simpson	50	0.88	0.04	50	0.92	0.03	9.00%	−1.12 [−1.54, −0.70]
Nita et al., 2025 [46]	Simpson	35	23.74	8.16	35	18.7	7.08	8.23%	0.65 [0.11, 1.18]
Chen et al., 2025 [34]	Simpson	35	0.26	0.16	30	0.24	0.19	8.55%	0.11 [−0.37, 0.60]
Wu et al., 2025 [45]	Simpson	87	0.9492	0.01	70	0.9477	0.011	9.67%	0.14 [−0.17, 0.46]
Wu et al., 2024 [41]	Simpson	182	0.95	0.017	120	0.95	0.018	10.12%	0.00 [−0.23, 0.23]
Zhu et al., 2017 [23]	Simpson	40	0.106	0.069	44	0.1	0.061	8.96%	0.09 [−0.34, 0.52]
Zhu et al.,2025 [43]	Simpson	36	0.96	0.016	55	0.955	0.011	8.99%	0.38 [−0.05, 0.80]
Yuan et al., 2019 [29]	Simpson	107	0.923	0.045	107	0.954	0.023	9.87%	−0.86 [−1.14, −0.58]
	**Total (95% CI)**	**704**			**631**			**100%**	**−0.03 [−0.39, 0.34]**

Legend: 1—N_SCZ, 2—mean_Simpson_SCZ, 3—SD_Simpson_SCZ, 4—N_HC, 5—mean_Simpson_HC, 6—SD_Simpson_HC, 7—Weight.

**Table 4:** Observed index.

Study	Alpha Index	1	2	3	4	5	6	7	Std. Mean Difference IV, Random, 95% CI
Shi et al., 2023 [10]	Observed	39	225.32	53.77	44	218.58	49.08	11.95%	0.13 [−0.30, 0.56]
Chen et al., 2024 [40]	Observed	30	278.6	67.54	36	295.04	49.41	10.27%	−0.28 [−0.77, 0.21]
Chen et al., 2021 [42]	Observed	63	186.01	63.43	40	192.28	37.14	13.19%	−0.11 [−0.51, 0.28]
Yan et al., 2022 [39]	Observed	50	238.21	80.67	50	416.06	333.15	12.89%	−0.73 [−1.13, −0.32]
Nita et al., 2025 [46]	Observed	35	86.78	21.32	35	96.42	47.68	9.34%	−0.26 [−0.78, 0.26]
Zhu et al., 2017 [23]	Observed	40	233.37	258.23	44	216.65	262.39	12.06%	0.06 [−0.36, 0.49]
Li et al., 2021 [35]	Observed	38	95.78	32.54	38	103.47	34.11	11.32%	−0.23 [−0.68, 0.22]
Yuan et al., 2019 [29]	Observed	107	451.17	107.03	107	453.01	71.73	18.98%	−0.02 [−0.29, 0.25]
	**Total (95% CI)**	**402**			**394**			**100%**	**−0.17 [−0.40, 0.06]**

Legend: 1—N_SCZ, 2—mean_Observed_SCZ, 3—SD_Observed_SCZ, 4—N_HC, 5—mean_Observed_HC, 6—SD_Observed_HC, 7—Weight.

**Table 5:** ACE index.

Study	Alpha Index	1	2	3	4	5	6	7	Std. Mean Difference IV, Random, 95% CI
Shi et al., 2023 [10]	ACE	39	278.44	64.44	44	279.42	69.61	14.34%	−0.01 [−0.45, 0.42]
Chen et al., 2024 [40]	ACE	30	364.99	89.16	36	358.65	48.44	14.19%	0.09 [−0.40, 0.57]
Chen et al., 2021 [42]	ACE	63	211.32	52.2	40	217.08	34.45	14.43%	−0.12 [−0.52, 0.27]
Chen et al., 2025 [34]	ACE	35	275.86	101.97	30	272.91	162.56	14.18%	0.02 [−0.47, 0.51]
Zhu et al., 2017 [23]	ACE	40	315.82	79.27	44	331.99	68.66	14.35%	−0.22 [−0.65, 0.21]
Zhu et al., 2025 [43]	ACE	36	8795.91	81.64	55	9040.81	81.63	13.81%	−2.97 [−3.58, −2.37]
Yuan et al., 2019 [29]	ACE	107	583.94	135.45	107	566.45	74.21	14.71%	0.16 [−0.11, 0.43]
	**Total (95% CI)**	**402**			**394**			**100%**	**−0.17 [−0.40, 0.06]**

Legend: 1—N_SCZ, 2—mean_ACE_SCZ, 3—SD_ACE_SCZ, 4—N_HC, 5—mean_ACE_HC, 6—SD_ACE_HC, 7—Weight.

**Table 6:** Chao1 index.

Study	Alpha Index	1	2	3	4	5	6	7	Std. Mean Difference IV, Random, 95% CI
Chen et al., 2024 [40]	Chao1	30	364.7	83.4	36	365.97	58.13	9.24%	−0.02 [−0.50, 0.47]
Chen et al., 2021 [42]	Chao1	63	215.54	55.54	40	225.92	49.44	13.75%	−0.19 [−0.59, 0.20]
Chen et al., 2025 [34]	Chao1	35	283.25	106.02	30	272.77	78.54	9.11%	0.11 [−0.38, 0.60]
Zhu et al., 2017 [23]	Chao1	40	319.27	85.95	44	334.28	54.11	11.77%	−0.21 [−0.64, 0.22]
Tao et al., 2025 [44]	Chao1	49	910	88.73	49	921	111.35	13.81%	−0.11 [−0.50, 0.29]
Zhu et al., 2025 [43]	Chao1	36	8858.1	263.52	55	8939.18	243.25	12.14%	−0.32 [−0.74, 0.10]
Yuan et al., 2019 [29]	Chao1	107	567.85	137.18	107	558.69	89.44	30.18%	0.08 [−0.19, 0.35]
	**Total (95% CI)**	**360**			**361**			**100%**	**−0.07 [−0.22, 0.08]**

1—N_SCZ, 2—mean_Chao1_SCZ, 3—SD_Chao1_SCZ, 4—N_HC, 5-mean_Chao1_HC, 6—SD_Chao1_HC, 7—Weight.

## Data Availability

Data is contained within the article or Supplementary Material.

## Supplementary Materials

The following supporting information can be downloaded at: https://www.mdpi.com/article/10.3390/ijms27104606/s1.
