# Supplementary material for: Gut Microbiota in Schizophrenia: Taxonomic Shifts, Beta- Diversity Alterations, and Biomarker Potential: A Systematic Review
Source: Int J Mol Sci. 2026 May 21;27(10):4606. doi: 10.3390/ijms27104606 (PMC13207661; doi:10.3390/ijms27104606)
Supplement: Supplementary file 1 [file ijms-27-04606-s001.zip › ijms-4257823-supplementary.pdf]

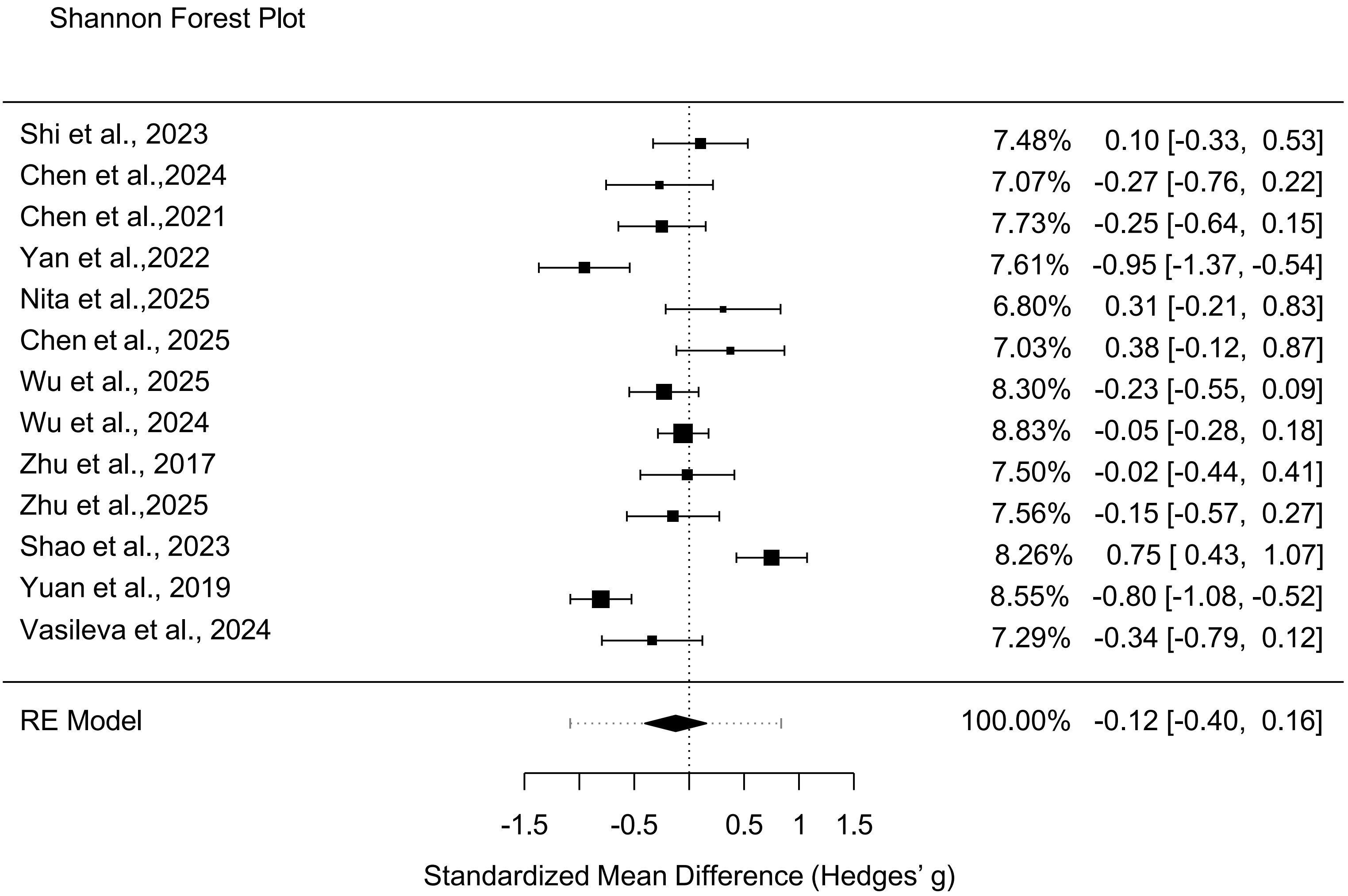
Shannon Forest Plot
Shi et al., 2023 7.48% 0.10 [-0.33, 0.53]
Chen et al.,2024 7.07% -0.27 [-0.76, 0.22]
Chen et al.,2021 7.73% -0.25 [-0.64, 0.15]
Yan et al.,2022 7.61% -0.95 [-1.37, -0.54]
Nita et al.,2025 6.80% 0.31 [-0.21, 0.83]
Chen et al., 2025 7.03% 0.38 [-0.12, 0.87]
Wu et al., 2025 8.30% -0.23 [-0.55, 0.09]
Wu et al., 2024 8.83% -0.05 [-0.28, 0.18]
Zhu et al., 2017 7.50% -0.02 [-0.44, 0.41]
Zhu et al.,2025 7.56% -0.15 [-0.57, 0.27]
Shao et al., 2023 8.26% 0.75 [0.43, 1.07]
Yuan et al., 2019 8.55% -0.80 [-1.08, -0.52]
Vasileva et al., 2024 7.29% -0.34 [-0.79, 0.12]
RE Model 100.00% -0.12 [-0.40, 0.16]
-1.5
-0.5
0.5
1
1.5
Standardized Mean Difference (Hedges' g)

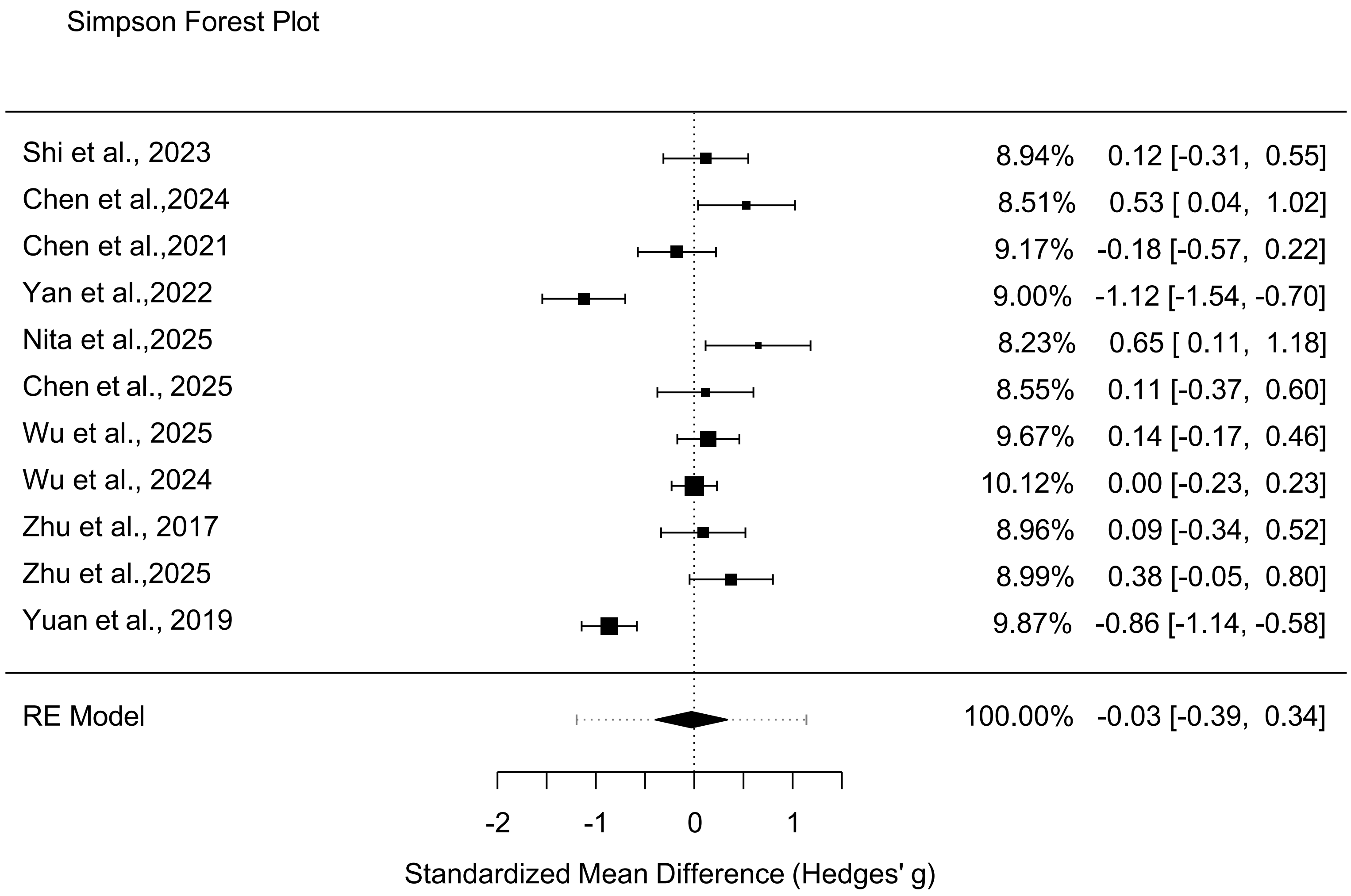
Simpson Forest Plot
Shi et al., 2023 8.94% 0.12 [-0.31, 0.55]
Chen et al.,2024 8.51% 0.53 [0.04, 1.02]
Chen et al.,2021 9.17% -0.18 [-0.57, 0.22]
Yan et al.,2022 9.00% -1.12 [-1.54, -0.70]
Nita et al.,2025 8.23% 0.65 [0.11, 1.18]
Chen et al., 2025 8.55% 0.11 [-0.37, 0.60]
Wu et al., 2025 9.67% 0.14 [-0.17, 0.46]
Wu et al., 2024 10.12% 0.00 [-0.23, 0.23]
Zhu et al., 2017 8.96% 0.09 [-0.34, 0.52]
Zhu et al.,2025 8.99% 0.38 [-0.05, 0.80]
Yuan et al., 2019 9.87% -0.86 [-1.14, -0.58]
RE Model 100.00% -0.03 [-0.39, 0.34]
-2
-1
0
1
Standardized Mean Difference (Hedges' g)

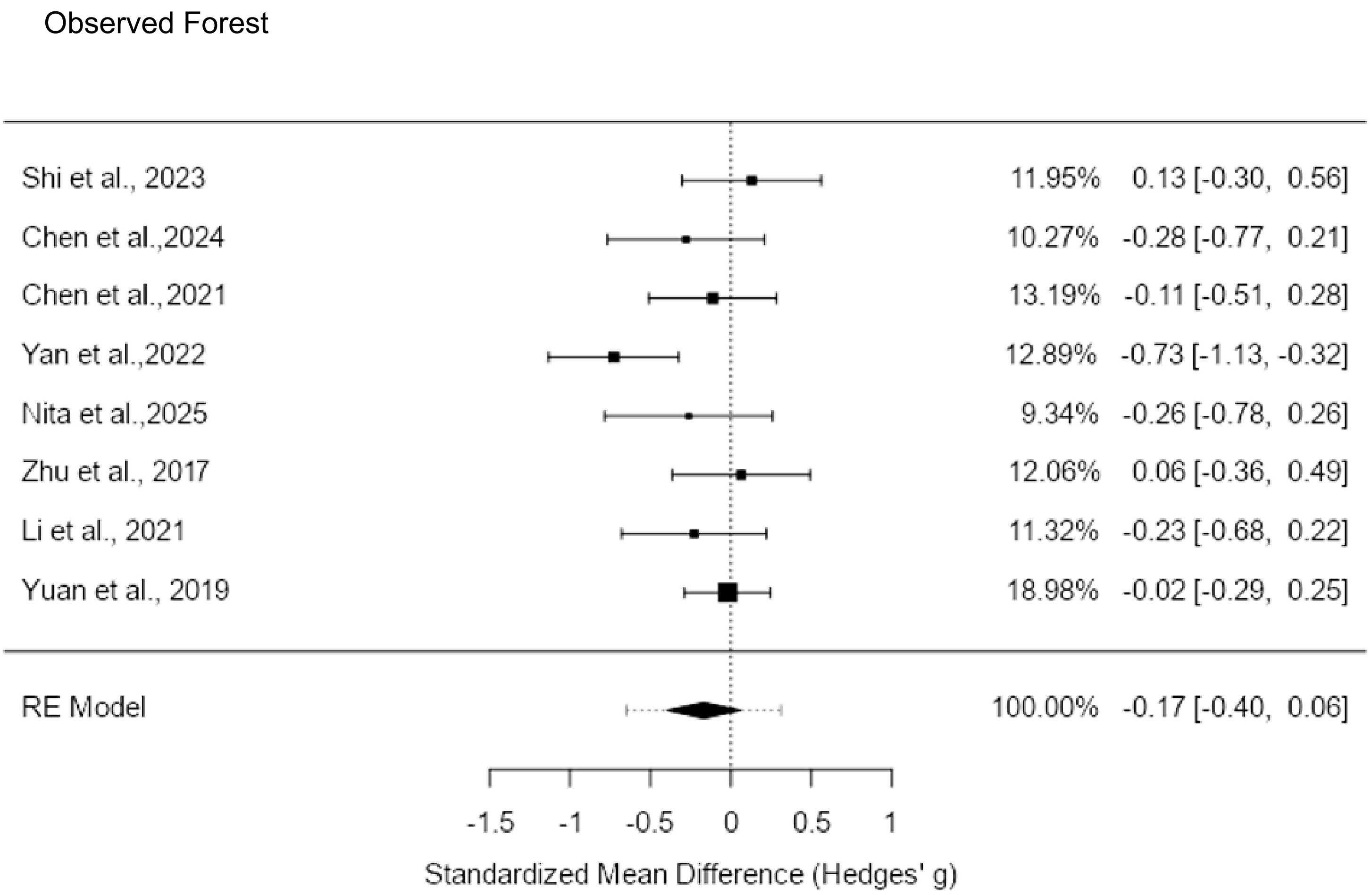

Observed Forest
Shi et al., 2023
Chen et al.,2024
Chen et al., 2021
Yan et al.,2022
Nita et al.,2025
Zhu et al., 2017
Li et al., 2021
Yuan et al., 2019
RE Model
11.95% 0.13 [-0.30, 0.56]
10.27% -0.28 [-0.77, 0.21]
13.19% -0.11 [-0.51, 0.28]
12.89% -0.73 [-1.13, -0.32]
9.34% -0.26 [-0.78, 0.26]
12.06% 0.06 [-0.36, 0.49]
11.32% -0.23 [-0.68, 0.22]
18.98% -0.02 [-0.29, 0.25]
100.00% -0.17 [-0.40, 0.06]
-1.5
-1
-0.5
0
0.5
1
Standardized Mean Difference (Hedges' g)

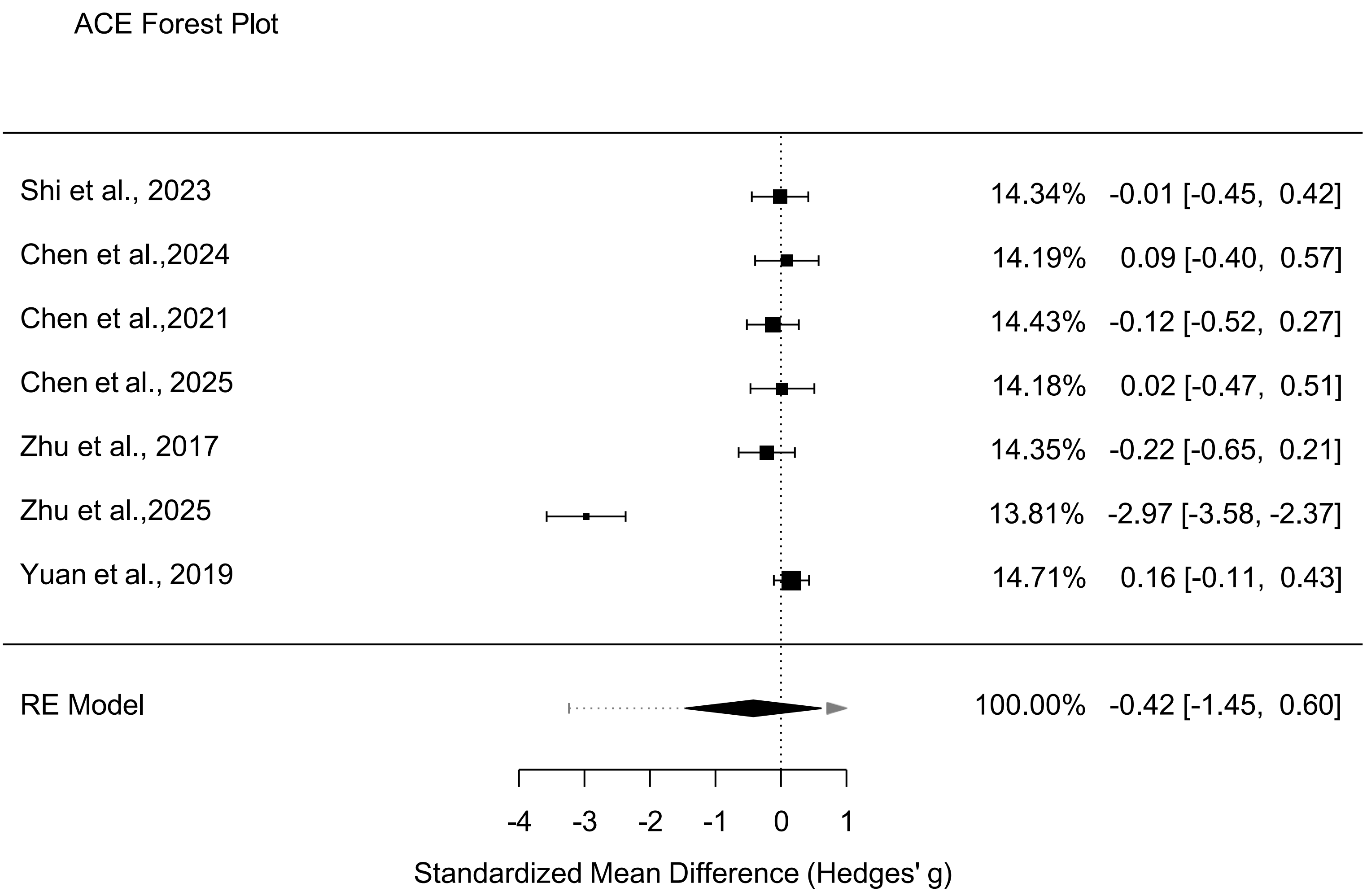
ACE Forest Plot
Shi et al., 2023
Chen et al.,2024
Chen et al.,2021
Chen et al., 2025
Zhu et al., 2017
Zhu et al.,2025
Yuan et al., 2019
RE Model
14.34% -0.01 [-0.45, 0.42]
14.19% 0.09 [-0.40, 0.57]
14.43% -0.12 [-0.52, 0.27]
14.18% 0.02 [-0.47, 0.51]
14.35% -0.22 [-0.65, 0.21]
13.81% -2.97 [-3.58, -2.37]
14.71% 0.16 [-0.11, 0.43]
100.00% -0.42 [-1.45, 0.60]
-4
-3
-2
-1
0
1
Standardized Mean Difference (Hedges' g)

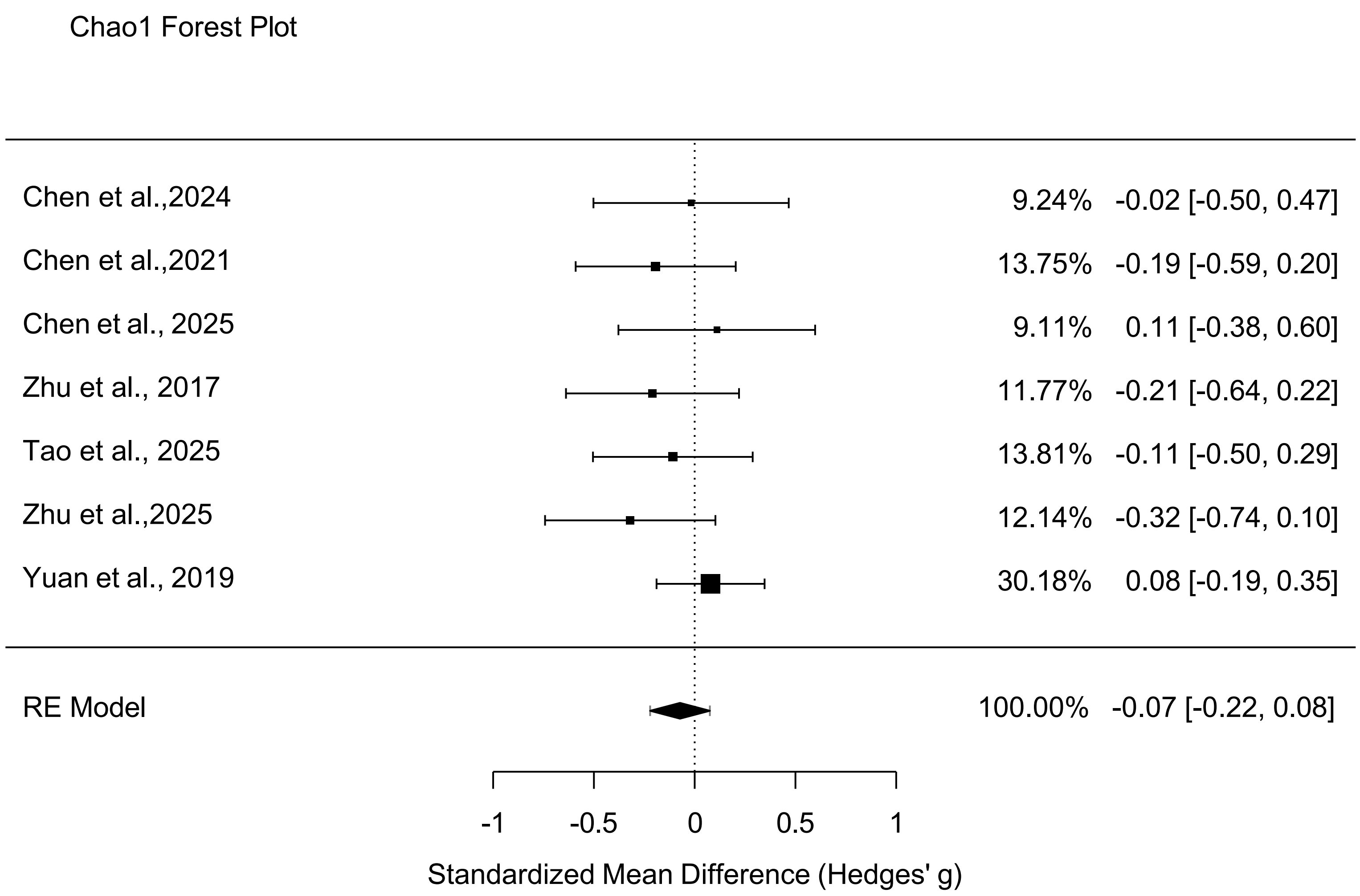
Chao1 Forest Plot
Chen et al.,2024
9.24%
-0.02 [-0.50, 0.47]
Chen et al.,2021
13.75%
-0.19 [-0.59, 0.20]
Chen et al., 2025
9.11%
0.11 [-0.38, 0.60]
Zhu et al., 2017
11.77%
-0.21 [-0.64, 0.22]
Tao et al., 2025
13.81%
-0.11 [-0.50, 0.29]
Zhu et al.,2025
12.14%
-0.32 [-0.74, 0.10]
Yuan et al., 2019
30.18%
0.08 [-0.19, 0.35]
RE Model
100.00%
-0.07 [-0.22, 0.08]
-1
-0.5
0
0.5
1
Standardized Mean Difference (Hedges' g)
